# Bovine three-portion pericardial patch for reconstruction of the aorto-mitral curtain in infective endocarditis

**DOI:** 10.1186/s40792-018-0558-5

**Published:** 2019-01-04

**Authors:** Atsushi Hiromoto, Shun-Ichiro Sakamoto, Yasuo Miyagi, Takashi Nitta

**Affiliations:** Department of Cardiovascular Surgery, Nippon Medical School, 1-1-5 Sendagi, Bunkyo-ku, Tokyo, 113-8603 Japan

**Keywords:** Infective endocarditis, Aorto-mitral curtain, Patch repair

## Abstract

**Background:**

Surgery for infective endocarditis involving the aorto-mitral curtain (AMC) is challenging and requires extensive incisions and complex reconstruction procedures. However, in patients with preserved aortic annulus, reconstruction of the AMC is possible using a simple technique with limited incisions.

**Case presentation:**

A handmade bovine three-portion pericardial patch was used to reconstruct the AMC in a patient with severe endocarditis requiring double valve replacement; the technique allowed for steady anchorage of prosthetic valves without additional incisions other than conventional aortotomy and atriotomy. Postoperative echocardiography revealed normal cardiac function and no significant perivalvular leakage. The patient displayed complete recovery and was discharged on postoperative day 33. The patient was symptom-free at his 1-year follow-up and displayed normal laboratory and echocardiographic findings.

**Conclusion:**

The bovine three-portion pericardial patch is useful for reconstructing the AMC in patients with infective endocarditis accompanied by preserved aortic annulus.

## Background

Involvement of the aorto-mitral curtain (AMC) is uncommon in patients with infective endocarditis. Once it is affected, the procedure for double valve replacement becomes more complex as it necessitates reconstruction of the AMC. This surgery is challenging and is associated with a high mortality rate (20–30%) [1–3]. In contrast, in patients with preserved aortic annulus, it is possible to reconstruct the AMC using a simple technique requiring limited incisions. Here, we report the surgical case of a patient with infective endocarditis with AMC involvement who was treated using the conventional approach.

## Case presentation

**Fig. 1:**
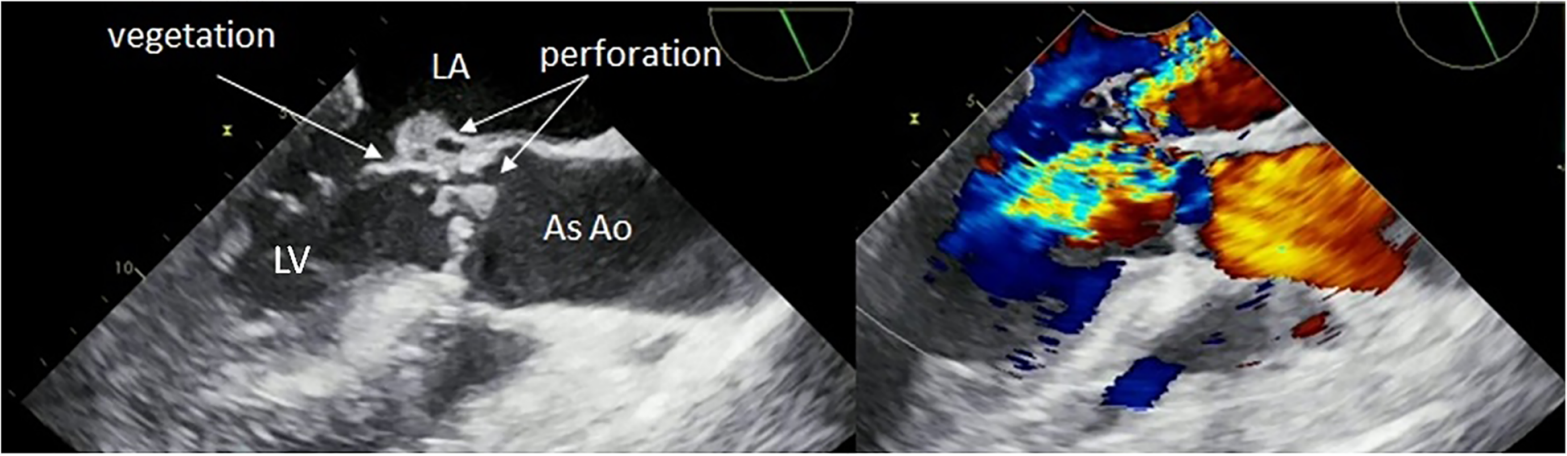
Three-chamber transoesophageal echocardiographic view reveals vegetation (size, 25 × 15 mm) attached to the anterior leaflet of the mitral valve. Color flow imaging reveals severe insufficiency of both the aortic and mitral valves with perforation in the AMC and at the base of the non-coronary cusp. LV left ventricle, LA left atrium, AsAo ascending aorta, AMC aorto-mitral curtain

A 57-year-old man was admitted to the hospital due to hyperleukocytosis. Echocardiography revealed irregularly shaped vegetation (size, 25 × 15 mm) attached to the anterior leaflet of the mitral valve. The vegetation exhibited oscillation and was connected to the thickened aortic valve. Color flow imaging showed severe insufficiency of both the aortic and mitral valves with perforation in the AMC (Fig. 1). Chest X-ray revealed bilateral lung congestion due to acute heart failure. Therefore, emergency surgery was indicated.

The heart was approached via median full sternotomy. An oblique incision was made in the ascending aorta under conditions of cardiac arrest. The aortic valve was bicuspid (type 1). Vegetation was observed at the non-coronary cusp, extending to the AMC. The mitral valve was exposed via the superior trans-septal approach. The anterior leaflet was thickened and had attached vegetation. Debridement of the infected tissue led to a defect in the middle portion of the anterior mitral annulus, AMC, and non-coronary cusp.

**Fig. 2:**
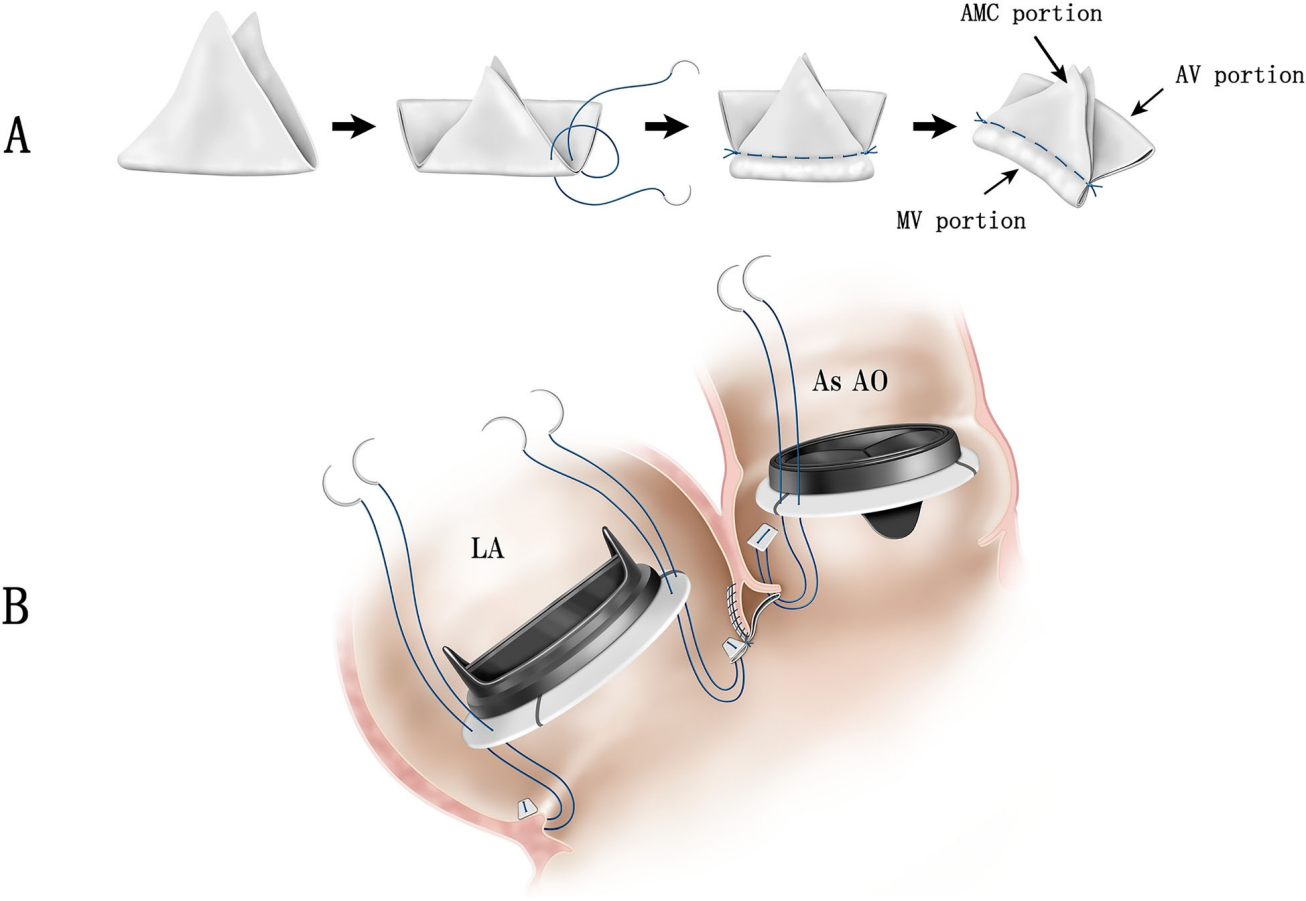
**a** Schematic illustration demonstrating the creation of a three-portion patch using a bovine pericardial patch. AMC aorto-mitral curtain, MV mitral valve, AV aortic valve. **b** Schematic illustration of double valve implantation in both the aortic and mitral annuli reconstructed with a three-portion patch. LA left atrium, AsAo ascending aorta

For reconstructing the defective parts, a glutaraldehyde-treated bovine pericardial patch (Model 4700, Edwards Lifesciences, Irvine, CA, USA) was folded to make a three-portion patch (Fig. 1a). The triangular portion (AMC portion) of two pericardial patches was sutured to the AMC remnant using continuous sutures. Pledgeted everted mattress sutures were placed around the mitral annulus, and the anterior rim was reconstructed with the pericardial patch (MV portion). A 23-mm mechanical valve (Abbott Laboratories, Chicago, IL, USA) was tied down in the intra-annular position of the aortic annulus in a manner wherein the sutures pass through the aortic annulus and the rectangular portion (AV portion) of the pericardial patch. Finally, a 28-mm mechanical valve (Abbott Laboratories, Chicago, IL, USA) was tied down in the mitral annulus (Fig. 2b).

*Cardiobacterium valvarum* was isolated on blood culture. Vancomycin and ceftriaxone were intravenously administered for 4 weeks postoperatively. Postoperative echocardiography revealed normal cardiac function with no significant perivalvular leakage. The patient displayed complete recovery and was discharged on postoperative day 33. The patient was symptom-free at his 1-year follow-up and exhibited normal laboratory and echocardiographic findings.

## Discussion

Several studies have reported on the most appropriate AMC reconstruction technique depending on the extent of involvement. For example, David et al. reported a technique for patch reconstruction of the anterior mitral annulus, left ventricular outflow tract, and left atrial roof in patients with an infected AMC [1]. Davierwala et al. used an aortic prosthesis or aortic root conduit in patients with more extensive aortic annular involvement [3]. Moreover, Tedoriya et al. reported a handmade aorto-mitral bioprosthetic valve created using two bioprosthetic valves and Dacron knitted fabric for reconstructing the AMC after extensive debridement [4]. These techniques require complex incisions in addition to routine aortotomy and atriotomy to expose the aortic and mitral valves.

In our case, aortotomy was performed to observe the aortic valve because the periannular tissue damage was not clearly identified via both preoperative and intraoperative echocardiography. In patients with involvement of the aortic annulus, annular incision using the Manouguian technique might be an appropriate add-on approach to mitral valve surgery [5]. However, this approach inevitably requires double valve replacement on the single layer of the pericardial patch. The advantage of our technique is that it allows reconstruction of the AMC without any additional incisions other than aortotomy and atriotomy; this facilitates the repair of the mitral valve or the implantation of two prosthetic valves in the natural annular position without excessive suture tension.

Use of the bovine pericardial patch is helpful in surgery for active infective endocarditis. Shinn et al. reported the use of a pericardial patch for reconstruction of the aortic annulus, AMC, and mitral annulus in 57 patients. They reported satisfactory outcomes at mid-term follow-up, thereby indicating the safety and durability of such patches [6]. However, complications associated with extensive use of the pericardium, such as valve dehiscence, perivalvular leakage, and pseudo-aneurysms, have been reported within the first year of surgery [3]. In our technique, the three-portion patch was created as a double layer. The shape of each patch portion was designed considering the suture line for the reconstruction of the AMC. The AMC patch portion was fitted in the triangular area surrounded by the left and right trigone and the mitral annulus. The aortic patch portion was rectangular and was horizontally sutured to the aortic annulus. We believe that this minimum usage of the double layer patch and the modified design for the AMC will render the mitral patch portion more durable and allow for anchorage of the prosthetic valve in patients requiring debridement of the AMC. In contrast, calcification of the pericardial patch has been reported particularly when used in the posterior rim of the mitral annuls. Dacron polyester fabric is an alternative material that may be preferable in this situation [7]. Further follow-up is required to ascertain the long-term durability of the pericardial patch.

## Conclusion

The bovine three-portion pericardial patch is useful for the reconstruction of the AMC in patients with infective endocarditis with preserved aortic annulus.

## Abbreviations

AMC: Aorto-mitral curtain
AV: Aortic valve
MV: Mitral valve
